# Metabolite Signatures and Particle Size as Determinants of Anti-Inflammatory and Gastrointestinal Smooth Muscle Modulation by *Chlorella vulgaris*

**DOI:** 10.3390/foods14193319

**Published:** 2025-09-25

**Authors:** Natalina Panova, Anelia Gerasimova, Mina Todorova, Mina Pencheva, Ivayla Dincheva, Daniela Batovska, Vera Gledacheva, Valeri Slavchev, Iliyana Stefanova, Stoyanka Nikolova, Irena Mincheva, Magdalena Szechyńska-Hebda, Krastena Nikolova

**Affiliations:** 1 Department of Physics and Biophysics, Faculty of Pharmacy, Medical University of Varna, 84 Tzar Osvoboditel, 9000 Varna, Bulgaria; panova@mu-varna.bg; 2 Department of Chemistry, Faculty of Pharmacy, Medical University of Varna, 84 Tzar Osvoboditel, 9000 Varna, Bulgaria; anelia.gerasimova@mu-varna.bg; 3 Department of Organic Chemistry, Faculty of Chemistry, University of Plovdiv, 4000 Plovdiv, Bulgaria; minatodorova@uni-plovdiv.bg (M.T.); tanya@uni-plovdiv.bg (S.N.); 4 Department of Medical Physics and Biophysics, Faculty of Pharmacy, Medical University of Plovdiv, 4002 Plovdiv, Bulgaria; mina.pencheva@mu-plovdiv.bg (M.P.); vera.gledacheva@mu-plovdiv.bg (V.G.); valeri.slavchev@mu-plovdiv.bg (V.S.); iliyana.stefanova@mu-plovdiv.bg (I.S.); 5 Department of Agrobiotechnologies, AgroBio Institute, Agricultural Academy, 8 Dragan Tsankov Blvd., 1164 Sofia, Bulgaria; iva.dincheva@yahoo.com; 6 Institute of Chemical Engineering, Bulgarian Academy of Sciences, Acad. G. Bonchev Str., Bl. 103, 1113 Sofia, Bulgaria; irenamincheva@gmail.com; 7 Institute of Electronics, Bulgarian Academy of Sciences, 72 Tzarigradsko Chaussee Blvd., 1784 Sofia, Bulgaria; 8 Polish Academy of Sciences, W. Szafer Institute of Botany, 31-512 Krakow, Poland; m.szechynska-hebda@botany.pl

**Keywords:** albumin denaturation, functional foods, gastric smooth muscle, GC–MS profiling, IL-1β, L-type calcium channels, microalgae, muscarinic acetylcholine receptors, nNOS, particle size

## Abstract

*Chlorella vulgaris* is a nutrient-dense microalga with recognized antioxidant, anti-inflammatory, and metabolic regulatory properties, making it an attractive candidate for functional food applications. In such contexts, both chemical composition and particle size can influence dispersibility, bioactive release, and physiological effects. In this study, two commercial *C. vulgaris* powders from India (Sample 1) and the UK (Sample 2) were compared with respect to particle size, metabolite composition, and biological activity. Sample 1 exhibited finer particles, while Sample 2 was coarser. GC–MS profiling revealed distinct compositional differences: Sample 1 displayed a higher relative abundance of saturated fatty acids, β-sitosterol, β-amyrin, and glucitol, whereas Sample 2 contained higher levels of unsaturated fatty acids, betulin, salicylic acid, and specific carbohydrates. In vitro assays showed stronger inhibition of albumin denaturation by Sample 1 compared with Sample 2 and prednisolone. Ex vivo tests indicated that both samples induced tonic contraction of gastric smooth muscle through muscarinic acetylcholine receptors (mAChRs) and L-type calcium channels, as evidenced by the marked reduction in responses after atropine and verapamil treatment, with Sample 1 producing a more pronounced effect. Immunohistochemistry further demonstrated broader IL-1β upregulation with Sample 1 and localized nNOS modulation with Sample 2. Overall, the results demonstrate that the interplay between composition and particle size shapes the bioactivity of *C. vulgaris*, supporting its targeted use in digestive, neuroimmune, and cardiometabolic health.

## 1. Introduction

*Chlorella vulgaris* (phylum Chlorophyta; family *Chlorellaceae*) is a unicellular microalga widely recognized as a source of bioactive compounds for nutraceuticals, including functional foods and dietary supplements [1,2]. It occurs in freshwater, marine, and terrestrial ecosystems and is characterized by rapid growth and tolerance to environmental stressors such as elevated CO_2_, nutrient limitation, and temperature fluctuations. Its scalability and high biomass productivity make it an attractive resource for environmentally friendly food production and biotechnological applications [3,4,5].

In addition to its cultivation advantages, *C. vulgaris* has a remarkable nutritional profile. On a dry-weight basis, it contains up to 60% protein with a balanced amino-acid composition, along with dietary fiber, vitamins (B, E, and D), chlorophyll, carotenoids such as lutein and zeaxanthin, and essential minerals including iron, zinc, and magnesium [1,6,7,8]. This composition underpins its widespread use in functional and fortified products, particularly in Asian and European markets, where it is commercialized as powders, capsules, and tablets [9].

Beyond its nutritional value, *C. vulgaris* demonstrates diverse biological activities relevant to human health, including antioxidant [10,11,12,13], anti-inflammatory [13,14,15,16], immunomodulatory [13,17,18,19], anticancer [11,20,21,22], and cardiometabolic effects [23,24,25,26]. These properties are attributed to a complex phytochemical repertoire that includes long-chain polyunsaturated fatty acids, phytosterols, triterpenoids, and polyphenols [8,25,26]. Recent findings further suggest that *C. vulgaris* polysaccharides can attenuate oxidative stress, while peptides can modulate NF-κB signaling and downregulate endothelin-1 (ET-1), a vasoconstrictive peptide implicated in vascular dysfunction [27,28]. Moreover, the alga may influence gut-microbiota composition, thereby supporting systemic metabolic and immune regulation [24,29,30,31].

Despite extensive evidence for systemic health benefits, the effects of *C. vulgaris* on gastrointestinal physiology—particularly smooth-muscle function—remain insufficiently understood. Supplementation has been shown to alleviate colitis in murine models through microbiota modulation and increased short-chain fatty acid production [29], while human trials report individualized metabolomic responses depending on the intestinal environment [32]. In vitro digestion studies further suggest that the rigid cell wall of *C. vulgaris* limits nutrient release, potentially influencing luminal signaling to the gut wall [33]. Gastrointestinal motility is governed by smooth-muscle contractility, which depends on cholinergic neurotransmission via muscarinic acetylcholine receptors (mAChRs; mainly M_2_ and M_3_ subtypes) and Ca^2+^ influx through L-type voltage-gated and receptor-operated TRPC4 channels [34]. Dysregulation of these pathways underlies disorders such as irritable bowel syndrome, functional dyspepsia, and post-infectious motility disturbances [35]. Natural compounds capable of modulating these mechanisms—particularly those antagonizing mAChRs or regulating Ca^2+^ signaling—are therefore of interest as functional-food ingredients with spasmolytic or neuromodulatory potential [36,37,38].

Emerging hypotheses suggest that the health-promoting effects of *C. vulgaris* may arise through systemic immunomodulation, such as regulatory T-cell activation and cytokine suppression, and/or through direct actions on neural and muscular targets. However, empirical support for the latter remains limited [29].

The present study therefore aimed to characterize the chemical composition and evaluate the anti-inflammatory and spasmolytic activities of two commercial *C. vulgaris* extracts, to assess their potential as functional-food ingredients for digestive health. Using GC–MS profiling, particle-size analysis, in vitro anti-denaturation assays, immunohistochemistry, and ex vivo contractility testing, we show that compositional and physical differences between the two products translate into distinct biological profiles. Specifically, one extract was associated with stronger anti-inflammatory effects and generalized immune activation, while the other demonstrated more balanced neuroimmune modulation with attenuated pro-inflammatory signaling. These findings highlight the importance of integrating chemical and physical characterization in defining the functional applications of *C. vulgaris*.

## 2. Materials and Methods

### 2.1. Sample Description

Two commercially available *C. vulgaris* powders were analyzed to compare their chemical composition and biological activities. Sample 1 and Sample 2 were selected to represent commercially relevant powders from different geographic and regulatory contexts, exhibiting differences in declared nutritional composition.

Sample 1—obtained from a biomarket in Plovdiv, Bulgaria. The label stated “produced in India” and “distributed by a company based in Berlin, Germany”. Declared nutritional composition per 100 g: total fat, 11.0 g (including 3.3 g monounsaturated fatty acids); carbohydrates, 15.0 g (of which sugars, 3.2 g); protein, 59.0 g; dietary fiber, 7.0 g.

Sample 2—purchased from a biomarket in Sofia, Bulgaria. The label indicated origin from the United Kingdom. Declared nutritional composition per 100 g: energy, 343 kcal; total fat, 7.8 g; carbohydrates, 6.9 g; protein, 57.2 g; dietary fiber, 16.1 g.

Both commercial powders were marketed in the EU as foods/food supplements and are therefore subject to Regulation (EC) No 178/2002 (General Food Law), Regulation (EC) No 852/2004 (food hygiene), Regulation (EU) No 1169/2011 (food information to consumers), and contaminant limits established by Regulation (EU) 2023/915. Chlorella sp. is considered not novel under Regulation (EU) 2015/2283 (as notified in the EU Novel Food consultation database). Manufacturers state compliance with HACCP and routine monitoring for heavy metals and microcystins. However, the specific cultivation, media composition, and processing details for these products were not disclosed. This uncertainty was intentionally included in our study design, allowing evaluation of products exactly as marketed rather than under idealized laboratory conditions. The difference in particle size is a key factor influencing dispersibility, bioactive release, and physiological effects, and may affect bioavailability, gastrointestinal tolerance, and potential interactions with medications, providing insights relevant to consumer health, food safety, and regulatory compliance. Correlating these physical properties with chemical differences and biological activity will provide mechanistic insight into functional outcomes.

### 2.2. Particle-Size Analysis

The particle-size distribution of the *C. vulgaris* powders was determined to evaluate their suitability for homogeneous dispersion in dietary supplement formulations. Measurements were performed in dry-dispersion mode using a Mastersizer 3000 laser-diffraction particle-size analyzer (Malvern Instruments Ltd., Worcestershire, UK) under standard operating conditions, with each sample analyzed in triplicate.

The surface area moment mean diameter (D[3,2]), and the volume moment mean diameter (D[4,3]) were calculated to assess the contributions of fine and coarse particles, respectively, according to Equation (1) (following ISO 13320 [39] and instrument guidance).

(1) $$D_{\left[3,2\right]}=\frac{\sum n_{i}d_{i}^{3}}{\sum n_{i}d_{i}^{2}}\;and\;D_{\left[4,3\right]}=\frac{\sum n_{i}d_{i}^{4}}{\sum n_{i}d_{i}^{3}}$$

In addition, particle-size percentiles Dv10, Dv50, and Dv90 were recorded, representing the diameters below which 10%, 50%, and 90% of the sample volume is contained.

### 2.3. GC–MS Sample Preparation and Analysis

Extraction and analysis of polar metabolites (organic acids, amino acids, sugars, sugar alcohols) and lipophilic compounds (fatty acids, sterols) were performed following Dincheva et al. [40] with minor modifications. Each *C. vulgaris* sample was homogenized in methanol using an Ultra-Turrax T25 (IKA, Staufen, Germany) at 10,000 rpm for 30 s. The homogenate was incubated in a TS-100 thermoshaker (Analytik Jena AG, Jena, Germany) at 4 °C and 120 rpm for 12 h, then centrifuged at 13,000 rpm for 10 min at 22 °C (Beckman Coulter, Brea, CA, USA). The supernatant was collected for GC–MS analysis.

GC–MS was carried out on a 7890A gas chromatograph coupled with a 5975C mass-selective detector (Agilent Technologies, Santa Clara, CA, USA) equipped with an HP-5 ms capillary column (30 m × 0.32 mm i.d., 0.25 µm film thickness, dimethylsiloxane stationary phase). The oven program was set from 60 °C (no hold) to 300 °C at 5 °C/min, with a final hold of 10 min. Helium was used as the carrier gas at a flow rate of 1.0 mL/min. Injector and detector temperatures were maintained at 250 °C.

Relative retention indices (RI) were calculated using an *n*-alkane mixture (C10–C40, Sigma-Aldrich, Tokyo, Japan) under identical conditions. Compound identification was based on RI matching with authentic standards and spectral comparison with the NIST 08 library [41], the Golm Metabolome Database (GMD) [42], and published data [40]. For compounds identified via libraries, only matches with ≥90% similarity and RI agreement within ±2% of reference values were accepted. Relative abundance of components refers to relative peak areas obtained under identical extraction and GC–MS conditions, and differences between samples that are expressed as log_2_ fold change values (log_2_ (Fold Change Sample 2/Sample 1).

### 2.4. Inhibition of Albumin Denaturation

Dry *C. vulgaris* powder was extracted with 80% methanol (1:10 *w*/*v*) in an ultrasonic bath at 40 °C for 40 min. The extract was filtered through Whatman filter paper [43], and the solvent was evaporated under reduced pressure using a Rotary Evaporator (BÜCHI Labortechnik AG, Flawil, Switzerland). The dried residue was reconstituted in dimethyl sulfoxide (DMSO) to a final concentration of 2.5 mg/mL.

Heat-induced serum albumin denaturation is a widely used in vitro proxy of anti-inflammatory potential because denatured proteins may act as autoantigens in inflammatory processes; compounds that inhibit denaturation are considered to exert membrane-stabilizing and anti-inflammatory effects under assay conditions. The anti-denaturation assay was performed according to Milusheva et al. [43,44] with slight modifications. Reaction mixtures contained: 0.5 mL of 5% human serum albumin (Albunorm 20, Octapharma AG, Brussels, Belgium), 0.2 mL of extract solution (2.5 mg/mL in DMSO), and 2.5 mL PBS (pH 6.3). Blanks received 2.5 mL PBS + 0.2 mL DMSO (without extract), while controls contained 0.5 mL albumin + 2.5 mL PBS (without extract or DMSO). All mixtures were incubated at 37 °C for 15 min, heated to 80 °C for 30 min, cooled for 5 min, and turbidity was measured at 660 nm using a Cary 60 UV-Vis spectrophotometer (Agilent Technologies, Santa Clara, CA, USA).

The percentage inhibition of protein denaturation (% IPD) was calculated according to Equation (2).

(2) $$\%\;IPD\;=\;\frac{\left(Abs\;control\;-Abs\;sample\right)}{Abs\;control}\times 100$$

The control represents 100% protein denaturation. Prednisolone (2.5 mg/mL) served as a positive control.

### 2.5. Spasmolytic Effect

#### 2.5.1. Ex Vivo Experiments

Male Wistar rats (3–4 months old) were obtained from the vivarium of the Medical University of Plovdiv, Bulgaria. The animals were maintained under controlled conditions (22 ± 2 °C, 12 h light/dark cycle, relative humidity of 50–60%) in individually ventilated cages with wood-chip bedding. They had ad libitum access to standard rodent chow and water. The enrichment was provided using nesting material and shelters to promote natural behaviors. Before tissue collection, animals were fasted for 12–16 h to empty the stomach while maintaining ad libitum access to water, in order to reduce variability in gastric contents and facilitate tissue preparation. After the fasting period, animals were humanely sacrificed following administration of an overdose of anesthetics, consisting of xylazine (2%, 10 mg/kg; Merck, Darmstadt, Germany) combined with ketamine (5%, 100 mg/kg; Merck, Darmstadt, Germany), delivered via intraperitoneal injection. All procedures complied with EU Directive 2010/63/EU and were approved by the institutional animal ethics committee. The experiments also adhered to the requirements of the Bulgarian legislation, namely the Law on the Protection of Animals (SG No. 13/2008, last amended SG No. 65/2020) and the Ordinance No. 20/01.11.2012 on the minimum requirements for the protection and welfare of experimental animals and the requirements for their use in experiments (No. 20/01.11.2012 г.), issued by the Ministry of Agriculture, Food and Forestry.

#### 2.5.2. Gastric Smooth Muscle Preparations and Evaluation of Spontaneous Contractile Activity

Following deep anesthesia, the stomach was excised via laparotomy, and longitudinal smooth muscle strips (~12–13 mm × ~1.0–1.1 mm) were isolated. Preparations were mounted in 15 mL organ baths containing Krebs solution (37 °C, aerated with 95% O_2_/5% CO_2_). One end of each strip was fixed to a stationary hook, and the opposite end was connected to an isometric force transducer integrated into a Radnoti 4-Unit Tissue Organ Bath System (Model 159920, Radnoti, Dublin, Ireland). Contractile activity was continuously recorded using a PowerLab data acquisition system (ADInstruments, Dunedin, New Zealand) coupled with LabChart software v8.1.30 (25-Jul-2024). and the Dose Response Add-On v2.6.1 (ADInstruments, Dunedin, New Zealand).

Tissues were equilibrated for 60 min with bath solution changes every 15 min. Relaxant effects were assessed after precontraction with 10^−6^ M acetylcholine (ACh) (Sigma-Aldrich, Darmstadt, Germany), while direct spasmogenic activity was evaluated by cumulative addition of the *C. vulgaris* extracts. Contractile force was expressed in milliNewtons (mN).

### 2.6. Immunohistochemistry

#### 2.6.1. Staining Protocol

Formalin-fixed, paraffin-embedded gastric tissue sections (5 µm) were deparaffinized, rehydrated, and immunostained for IL-1β and nNOS (Elabscience Biotechnology Inc., Houston, TX, USA; dilution 1:300; 60 min) using the Autostainer Link 48 with the EnVision FLEX detection system (Dako, Agilent Technologies Inc., Glostrup, Denmark). Mayer’s hematoxylin (Merck, Darmstadt, Germany) was applied for counterstaining. Images were acquired with a Leica DM1000 LED microscope equipped with an ICC50 W digital camera (Leica Microsystems GmbH, Wetzlar, Germany).

#### 2.6.2. Quantitative Analysis

Pixel intensity (0–256 AU) was measured in circular/longitudinal smooth muscle and myenteric plexus layers using LAS X softwarev4.7.2 (Leica Microsystems, Wetzlar, Germany). For each animal, five sections were analyzed with ≥50 measurements per section.

### 2.7. Statistical Analysis

Data are presented as mean ± SD. Analyses were performed using GraphPad Prism v8.0.1 (GraphPad Software, La Jolla, CA, USA) and IBM SPSS Statistics v23.0 (IBM Corp., Armonk, NY, USA). Immunohistochemistry results were evaluated using one-sample *t*-tests and Wilcoxon signed-rank tests, with significance at *p* < 0.001. Chemical composition and anti-inflammatory activity were analyzed using Duncan’s multiple range test to compare group means. Spasmolytic activity was assessed on tissue preparations, with *n* representing the number of preparations analyzed per experiment, and comparisons between two independent groups were conducted using independent samples *t*-tests, with *p* < 0.05 considered statistically significant.

## 3. Results

### 3.1. Particle Size and Distribution

**Figure 1:**
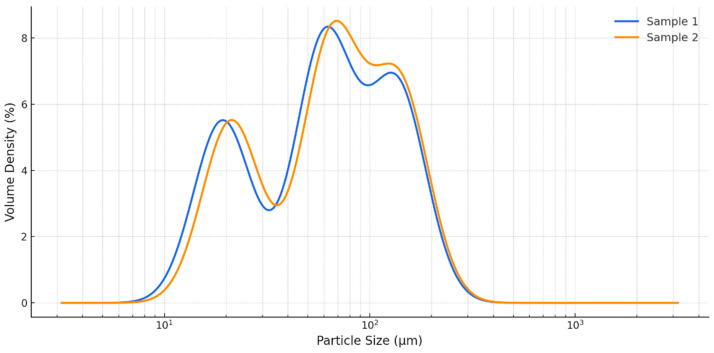
Particle size distribution profiles of dry *C. vulgaris* powders from India (Sample 1) and the UK (Sample 2).

Sample 2 (UK) exhibited slightly but significantly larger particle sizes for D[4,3], Dv50, and Dv90, indicating a shift toward coarser particles compared with Sample 1 (India) (Table 1). The distribution curves presented in Figure 1 confirmed this trend. No significant differences were observed for D[3,2] or Dv10.

### 3.2. Chemical Composition of the C. vulgaris Samples

**Figure 2:**
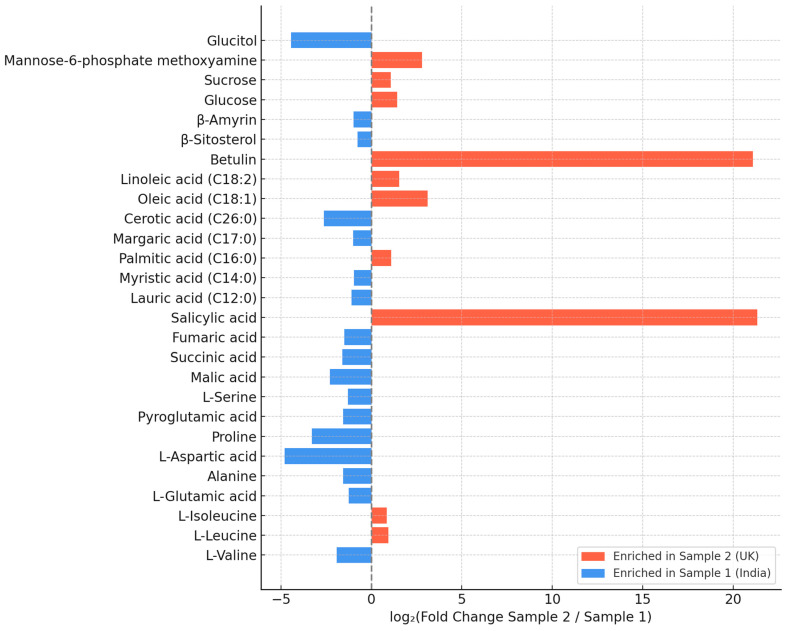
Fold change in significantly different metabolites between *C. vulgaris* samples from India (Sample 1) and the UK (Sample 2) as determined by GC–MS profiling. Fold change refers to the ratio of metabolite abundance between the two samples, expressed as an increase (>1) or decrease (<1) relative to the comparator. Full quantitative data, including retention times (RT) and retention indices (RI), are provided in Supplementary Tables S1–S3. Compound classes comprise amino acids and organic acids, fatty acids, triterpenoids, sterols, and carbohydrates.

GC–MS profiling of methanolic extracts revealed a diverse phytochemical composition, including amino acids, organic acids, fatty acids, triterpenoids, sterols, and carbohydrates. Full quantitative data are provided in Tables S1–S3, while Figure 2 presents only compounds that differed significantly (*p* < 0.05) between the two samples. The Indian product (Sample 1) showed comparatively a higher relative abundance of triterpenoids (β-amyrin), sterols (β-sitosterol), the sugar alcohol glucitol, and several amino acids (e.g., L-valine, L-aspartic acid, L-glutamic acid). By contrast, the UK product (Sample 2) contained markedly higher levels of unsaturated fatty acids (oleic and linoleic acids), betulin, and salicylic acid, along with elevated sucrose and mannose-6-phosphate. These compositional shifts highlight two distinct metabolic signatures that may underlie the sample-specific biological activities described in subsequent sections.

Amino and organic acids (Table S1). Sample 1 (India) contained several non-essential and conditionally essential amino acids, such as L-glutamic acid, L-aspartic acid, pyroglutamic acid, and alanine, together with malic, succinic, and fumaric acids. In contrast, Sample 2 (UK) contained higher levels of the essential branched-chain amino acids L-leucine and L-isoleucine, as well as salicylic acid.

Fatty acids, triterpenoids, and sterols (Table S2). Differences in lipid composition were also observed. Sample 1 contained higher amounts of saturated fatty acids, including cerotic, lauric, and myristic acids, together with elevated levels of β-amyrin and β-sitosterol. Sample 2 showed a higher relative abundance of palmitic, oleic, and linoleic acids and uniquely contained betulin.

Carbohydrates (Table S3). Sample 1 exhibited markedly higher levels of glucitol, whereas Sample 2 contained greater amounts of glucose, sucrose, and mannose-6-phosphate.

Overall, these compositional differences indicate distinct metabolic profiles between the two *C. vulgaris* products, which may underlie the sample-specific biological activities described in Section 3.3, Section 3.4 and Section 3.5.

### 3.3. In Vitro Inhibition of Albumin Denaturation

**Figure 3:**
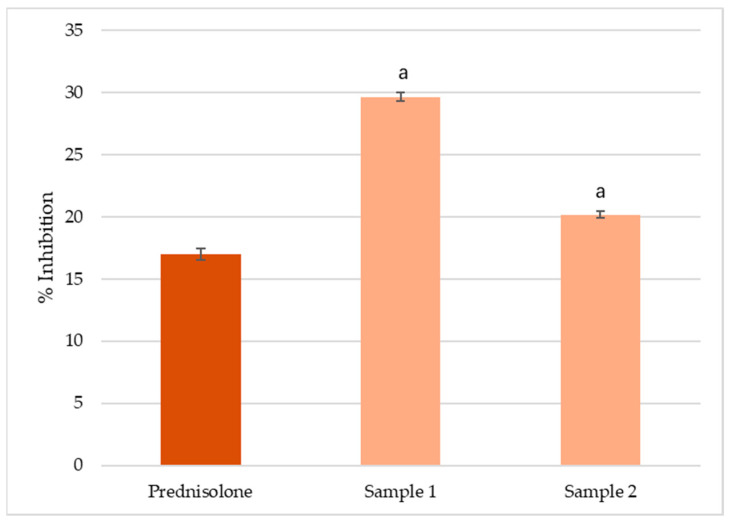
Inhibition of albumin denaturation by 80% methanol extracts of *C. vulgaris* from India (Sample 1) and the UK (Sample 2), compared with the reference anti-inflammatory drug prednisolone (2.5 mg/mL). Values are expressed as mean ± SD (*n* = 3). Letter **a** above the bars indicates statistically significant differences (*p* < 0.05, Duncan’s test).

The 80% methanol extract of *C. vulgaris* from India (Sample 1) exhibited the highest inhibition of albumin denaturation (30%), followed by the extract from the UK (Sample 2, 20%) and prednisolone (17%) (Figure 3). The differences among all treatments were statistically significant (*p* < 0.05), indicating that both *C. vulgaris* products possess measurable in vitro anti-inflammatory potential, with Sample 1 demonstrating the stronger effect.

### 3.4. Evaluation of Ex Vivo Spasmolytic Effect

#### 3.4.1. Effects on Spontaneous Smooth Muscle Contractile Activity

**Figure 4:**
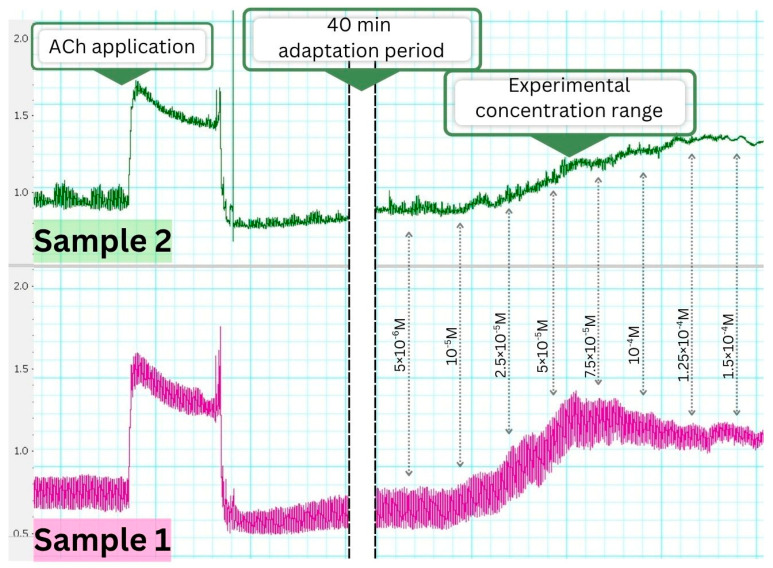
Representative tracings showing the effects of *C. vulgaris* from India (Sample 1) and the UK (Sample 2) on spontaneous contractile activity of isolated gastric smooth muscle strips from Wistar rats. Both extracts induced concentration-dependent tonic contractions without affecting the frequency of spontaneous rhythmic contractions.

Both methanolic extracts of *C. vulgaris* induced a tonic contractile effect on isolated gastric smooth muscle strips from Wistar rats, without altering the frequency of spontaneous rhythmic contractions within the tested concentration range (5 × 10^−6^ M to 1.5 × 10^−4^ M). The maximum tonic response was observed at 5 × 10^−5^ M for Sample 1 (India) and at 1 × 10^−4^ M for Sample 2 (UK), as illustrated by representative tracings (Figure 4).

#### 3.4.2. Pharmacological Modulation of the Contractile Response

To examine the pharmacodynamic profile of the spasmogenic activity, gastric smooth muscle strips were pretreated with acetylcholine (ACh), atropine, or verapamil. Neither extract interfered with ACh-induced contractions when applied afterward, indicating preserved receptor sensitivity. However, when ACh was applied before extract administration, both samples caused a marked reduction in the contractile response, suggesting possible post-receptor desensitization or inhibitory feedback.

Pretreatment with atropine significantly attenuated the contractile effect of both extracts, confirming muscarinic receptor involvement. Similarly, preincubation with verapamil reduced the tonic response, indicating the participation of L-type calcium channels. These interactions are summarized in Table 2.

### 3.5. Ex Vivo Immunohistochemical Analysis

**Figure 5:**
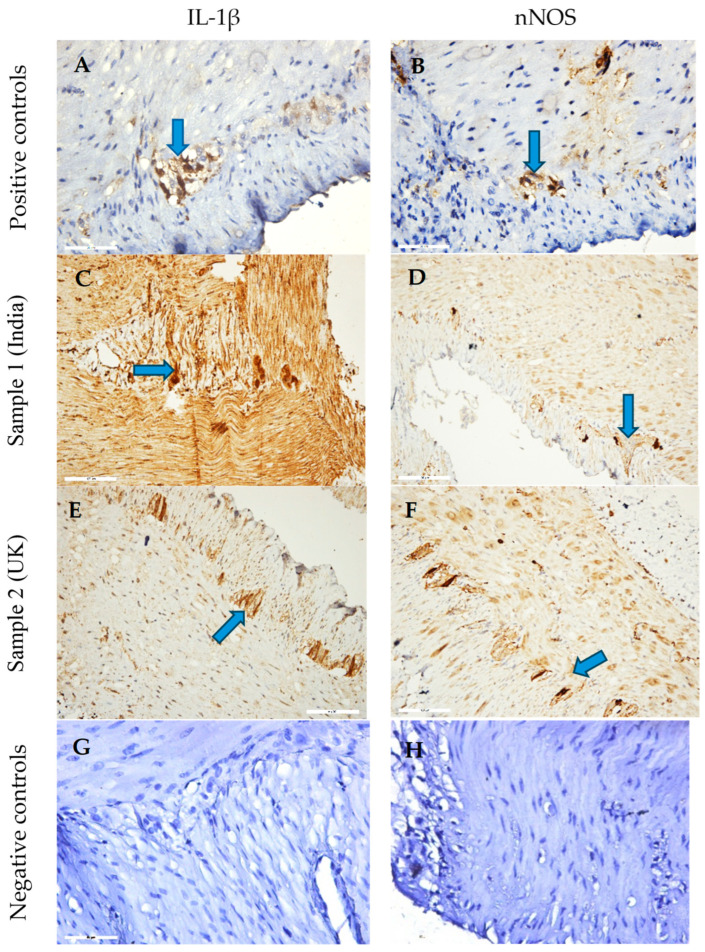
Representative immunohistochemical micrographs of gastric smooth muscle (SM) tissue from rats incubated with *C. vulgaris* methanol extracts. (**A**,**B**) Positive controls of the IL-1β and nNOS expression in the myenteric plexus (MP); (**C**,**D**) Sample 1 (India): strong IL-1β expression in both MP and SM; (**E**,**F**) Sample 2 (UK): weak to moderate expression of IL-1β and nNOS in MP and SM. Blue arrows indicate sites of immunoreactivity. (**G**,**H**) Negative controls of the IL-1β and nNOS, obtained by omitting primary antibodies in the tissue samples. Blue arrows indicate sites of immunoreactivity. Magnifications: ×200 (**A**,**B**,**E**,**F**) and ×400 (**C**,**D**,**G**,**H**).

Immunohistochemical staining revealed distinct patterns of IL-1β and nNOS expression in gastric tissues treated with *C. vulgaris* methanol extracts (Figure 5). Sample 1 (India) induced strong IL-1β immunoreactivity in both smooth muscle and the myenteric plexus, suggesting a pronounced pro-inflammatory response. Sample 2 (UK) showed moderate nNOS expression in neuronal structures and enhanced IL-1β staining in the myenteric plexus, indicative of localized inflammatory modulation and potential involvement of enteric neurons.

**Figure 6:**
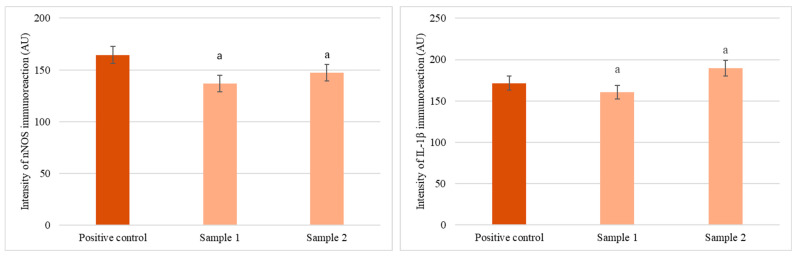
Quantitative morphometric analysis of nNOS (**left**) and IL-1β (**right**) immunoreactivity in gastric tissues following treatment with *C. vulgaris* methanol extracts. Values are expressed in arbitrary units (AU) as mean ± SD (*n* = 3). Letter **a** above bars indicates statistically significant differences (*p* < 0.001, Wilcoxon signed-rank tests).

Quantitative morphometric analysis (Figure 6) supported these observations. Sample 1 significantly reduced nNOS immunoreactivity compared with control (136.8 AU vs. 164.5 AU), whereas Sample 2 partially restored it (147.3 AU). IL-1β levels were highest in Sample 2-treated tissues (189.7 AU), followed by control (171.5 AU) and Sample 1 (160.5 AU).

These findings indicate that *C. vulgaris* extracts modulate nitrergic and inflammatory signaling in a sample-dependent manner, with Sample 2 associated with stronger IL-1β upregulation and milder suppression of nitrergic pathways.

## 4. Discussion

### 4.1. Particle Size and Functional Food Relevance

The physical characteristics of *C. vulgaris* powders are important determinants of functionality in food matrices, as they affect dispersion, solubility, and the release of bioactive compounds [45]. In this study, Sample 1 displayed finer particles, while Sample 2 showed significantly larger D[4,3], Dv50, and Dv90 values, indicating a shift toward coarser fractions. Finer particles, such as those in Sample 1, generally provide greater surface area and faster dissolution, which can enhance bioavailability and may partly explain its stronger in vitro anti-inflammatory activity. Conversely, the coarser profile of Sample 2 may slow dissolution but prolong release in the gastrointestinal tract, supporting more gradual and sustained functional effects [45,46]. Taken together, these observations highlight the need to integrate physical attributes with compositional profiling when evaluating the functional performance of *C. vulgaris* products.

### 4.2. Metabolite Profile and Functional Implications

In line with this, GC–MS profiling revealed pronounced compositional differences between the two samples, likely reflecting strain variation and cultivation conditions [47,48]. Sample 1 (India) showed a higher relative abundance of saturated fatty acids (cerotic, lauric, myristic), sterols (β-sitosterol), triterpenoids (β-amyrin), and the polyol glucitol, all linked to membrane stabilization, antimicrobial activity, and anti-inflammatory responses [49,50,51]. By contrast, Sample 2 (UK) contained higher levels of unsaturated fatty acids (oleic, linoleic), betulin, leucine, and carbohydrates such as mannose-6-phosphate and sucrose, associated with cardioprotective, metabolic, and neuroimmune regulation [5,52,53,54]. These differences in compound abundance reflect potential areas for further investigation of extract functional effects.

### 4.3. In Vitro Anti-Inflammatory Activity

Previous studies have shown that *C. vulgaris* extracts exert dose-dependent anti-inflammatory effects, with reported IC_50_ values typically ranging from 80 to 150 µg/mL depending on the fraction and assay employed [55,56]. In contrast, our assay was designed as a preliminary screening, using a fixed concentration (2.5 mg/mL) of the methanolic extracts. Both extracts inhibited albumin denaturation, with Sample 1 (India) showing the highest inhibition (30%), followed by Sample 2 (UK, 20%) and the reference drug prednisolone (17%). The stronger effect of Sample 1 is consistent with its higher relative abundance of sterols, triterpenoids, and polyols, compound classes known to stabilize protein conformation and suppress inflammatory mediator release [51,57,58,59]. These results indicate that multiple bioactive constituents likely act synergistically to prevent protein denaturation. While the findings support the anti-inflammatory potential of *C. vulgaris*, further studies with full concentration–response curves and IC_50_ determinations are required to validate and quantify these effects.

### 4.4. Smooth Muscle Contractility and Its Relation to Anti-Inflammatory Activity

The evaluation of gastric smooth muscle contractile activity provides a functional perspective on the biological effects of *C. vulgaris* extracts, complementing the chemical and in vitro anti-inflammatory data. Gastrointestinal smooth muscle contractility is tightly regulated by neuronal inputs, muscarinic receptors, and calcium-dependent signaling pathways, and it is frequently altered under inflammatory conditions [60,61,62]. Pro-inflammatory mediators, such as IL-1β, can sensitize smooth muscle and enteric neurons, leading to hypercontractility or dysmotility, which are hallmarks of gastrointestinal inflammatory disorders. In this study, Sample 1 (India) induced pronounced tonic contractions of isolated gastric smooth muscle, which corresponded with enhanced IL-1β expression and reduced nNOS immunoreactivity, suggesting broad pro-inflammatory activation alongside strong spasmogenic potential [63]. By contrast, Sample 2 (UK) elicited more moderate contractile effects, associated with localized IL-1β upregulation and partial restoration of nNOS, reflecting targeted neuroimmune modulation. These differences likely result from compositional divergence between the samples: saturated fatty acids, sterols, and triterpenoids in Sample 1 may promote membrane rigidity and facilitate calcium influx, enhancing contractility [64,65], whereas unsaturated fatty acids, betulin, and salicylic acid in Sample 2 may increase membrane fluidity and attenuate calcium-dependent contractions [66,67]. Thus, the assessment of smooth muscle contractility provides an integrative readout that links metabolite composition to functional outcomes. It allows the evaluation of both direct pharmacodynamic effects on muscle and indirect effects mediated by inflammatory signaling, highlighting how *C. vulgaris* extracts may modulate gastrointestinal motility under inflammatory or neuroimmune stress. Taken together, the stronger spasmogenic effect of Sample 1 compared with the more moderate activity of Sample 2 exemplifies how compositional divergence can translate into distinct functional outcomes. These findings emphasize the importance of combining chemical profiling, in vitro anti-inflammatory assays, and ex vivo functional analyses to comprehensively characterize the potential health benefits of nutraceutical preparations.

### 4.5. Contractile Activity and Calcium Modulation in Smooth Muscle

The divergent metabolite profiles were also reflected in the ex vivo contractility assays. Sample 1 induced stronger tonic and phasic contractions of gastric smooth muscle than Sample 2. In both cases, muscarinic receptor responsiveness was preserved, but the effects were markedly attenuated by atropine and verapamil, indicating the involvement of muscarinic acetylcholine receptors and L-type calcium channels [68].

The pronounced spasmogenic activity of Sample 1 may be attributed to its higher levels of saturated fatty acids, which can rigidify membranes, enhance depolarization, and facilitate voltage-dependent calcium influx [69,70,71], in combination with β-amyrin, reported to modulate ion-channel activity [72,73]. In contrast, the higher proportion of unsaturated fatty acids in Sample 2 may increase membrane fluidity and thereby dampen calcium-dependent contractile responses [69]. Additional compounds found in Sample 2, such as betulin and salicylic acid, may further attenuate contractility through interference with calcium influx and intracellular calcium signaling [74,75].

### 4.6. Immunohistochemical Evaluation of IL-1β and nNOS Expression

The contrasting metabolic and contractile characteristics of the two samples were mirrored in their immunohistochemical profiles. Sample 1 markedly upregulated IL-1β expression in both the smooth muscle (SM) layer and the myenteric plexus (MP), indicating broad pro-inflammatory activation spanning muscular and neuronal compartments. IL-1β is a central mediator that sensitizes nociceptive pathways, enhances excitatory synaptic transmission, and promotes leukocyte recruitment through NF-κB–dependent chemokine and adhesion molecule expression [76,77,78]. This effect may be associated with the higher levels of saturated fatty acids and sterols in Sample 1, as these lipids can activate Toll-like receptor 4 (TLR4) and promote clustering within cholesterol-rich membrane microdomains, thereby potentiating innate immune responses [79,80,81].

By contrast, Sample 2 displayed a more restricted pattern, with moderate nNOS expression within enteric ganglia and IL-1β upregulation limited to the MP, suggesting targeted neuroimmune modulation rather than generalized activation. This profile may reflect its enrichment in unsaturated fatty acids and betulin, both reported to suppress NF-κB signaling and attenuate cytokine production [82,83,84]. Since nNOS is the principal inhibitory neurotransmitter in enteric motor neurons, its partial restoration in Sample 2 may contribute to smooth muscle relaxation and maintenance of neuroimmune homeostasis [84].

### 4.7. Integrated Interpretation and Future Perspectives

Taken together, the results point to two distinct bioactivity profiles arising from compositional divergence between the samples. Sample 1 combined strong in vitro anti-inflammatory effects with generalized immune activation and pronounced spasmogenic potential, likely linked to its enrichment in β-sitosterol, β-amyrin, glucitol, and saturated fatty acids. The concomitant upregulation of IL-1β suggests potential application in gastrointestinal dysmotility with inflammatory components, although the pro-contractile profile warrants caution in hypermotility disorders.

In contrast, Sample 2 exhibited a more balanced bioactivity pattern, characterized by localized neuroimmune modulation—moderate nNOS induction, restricted IL-1β expression—and enrichment in unsaturated fatty acids and betulin. These attributes are consistent with NF-κB suppression and smooth muscle relaxation, supporting potential utility in neuroinflammatory conditions, irritable bowel syndromes with visceral hypersensitivity, or cardiovascular settings where vasorelaxation is desirable.

Physical attributes may further modulate these effects: finer particles in Sample 1 may enhance dispersibility and accelerate bioactive release, whereas the coarser profile of Sample 2 could favor slower dissolution and more sustained activity in gastrointestinal matrices.

Overall, the findings emphasize how lipid and triterpene composition, together with physical characteristics, shape functional outcomes of *C. vulgaris* products. Future studies should aim to delineate receptor-specific mechanisms, downstream inflammatory mediators, and interactions along the neuronal–glial–smooth muscle axis, complemented by standardized metabolite profiling, pharmacokinetic evaluation, and long-term safety assessment.

## 5. Conclusions

*Chlorella vulgaris* samples were selected to represent commercially relevant products from distinct geographic and regulatory contexts. The study demonstrates that both chemical composition and physical attributes, such as particle size, are critical determinants of the functional properties of different *C. vulgaris* extracts. Sample 1 (India) displayed greater relative levels of saturated fatty acids, β-sitosterol, and β-amyrin, was characterized by finer particle size, and elicited *strong* in vitro anti-inflammatory effects, broad immune activation, and pronounced spasmogenic responses. These properties have been previously associated with gastrointestinal dysmotility involving inflammatory components, although their relevance in hypermotility states remains unclear and requires further investigation [85,86,87]. In contrast, Sample 2 (UK), richer in unsaturated fatty acids, betulin, and salicylic acid and displaying a coarser particle profile, was associated with localized neuroimmune modulation, attenuated pro-inflammatory signaling, and minimal contractile stimulation, features that have been linked in previous studies to potential applications in neuroinflammatory and cardiovascular contexts where anti-inflammatory and vasorelaxant actions are relevant [81,88]. Overall, the findings highlight the importance of integrating compositional profiling with particle-size analysis to guide targeted applications of *C. vulgaris* in functional food formulations addressing digestive, neuroimmune, and cardiometabolic health. Correlation between chemical components and biological activity, the impact of particle size on dispersibility and bioactive release, and the implications for functional food products are all key points. Such correlations allow identification of food products from different sources that may be more suitable for specific applications, for example: modulation of gastrointestinal motility, neuroimmune support, or antioxidant effects. This provides practical value for functional foods, dietary supplements, and translational research. Further mechanistic studies, product standardization, and safety evaluation are needed to optimize its translational and nutraceutical potential.

## Figures and Tables

**Table 1:** Size distribution parameters of *C. vulgaris* dry samples.

Parameters	Sample 1 (India)	Sample 2 (UK)
D[3,2] (µm)	38.00 ± 0.68 ^a^	39.95 ± 0.67 ^a^
D[4,3] (µm)	70.20 ± 0.83 ^b^	72.54 ± 0.87 ^a^
Dv10 (µm)	19.20 ± 0.12 ^a^	21.20 ± 0.17 ^a^
Dv50 (µm)	60.40 ± 0.53 ^b^	65.40 ± 0.62 ^a^
Dv90 (µm)	136.00 ± 0.46 ^b^	140.00 ± 0.43 ^a^

Note: Means in a row with different superscript letters (a, b) differ significantly (*p* < 0.05; Duncan’s test); D[4,3]—volume-weighted mean diameter; Dv50/Dv90—particle size below which 50%/90% of particles fall; D[3,2]—surface-weighted mean diameter; Dv10—size below which 10% of particles fall.

**Table 2:** Effects of *C. vulgaris* methanolic extracts (Sample 1, India; Sample 2, UK) and reference agents (ACh, atropine, verapamil) on tonic contractile response (TCR), amplitude of spontaneous contractions (ASC), and frequency of spontaneous muscle contractions (FSMC) in isolated gastric smooth muscle strips from Wistar rats.

Pharmacological Agents	TCR, mN	ASC, mN	FSMC, n/min	*p*
Basal spontaneous contractile activity	2.07 ± 0.05	1.53 ± 0.17	5.00 ± 0.30	-
ACh	5.16 ± 0.05 ^a^	2.27 ± 0.20 ^a^	4.95 ± 0.03	0.01
Sample 2	4.17 ± 0.10 ^a^	2.15 ± 0.09 ^a^	4.88 ± 0.17	0.01
Sample 1	3.50 ± 0.13 ^a^	1.98 ± 0.05 ^a^	5.03 ± 0.09	0.01
Sample 2 + ACh	4.97 ± 0.10	1.96 ± 0.09	4.87 ± 0.11	0.06
Sample 1 + ACh	5.07 ± 0.12	2.07 ± 0.05	4.89 ± 0.22	0.06
ACh + Sample 2	3.49 ± 0.18 ^b^	0.60 ± 0.01 ^b^	5.10 ± 0.21	0.04
ACh + Sample 1	2.71 ± 0.09 ^b^	0.43 ± 0.07 ^b^	5.06 ± 0.02	0.03
Atropine	2.01 ± 0.05	1.99 ± 0.05	4.94 ± 0.04	0.06
Atropine + ACh	1.96 ± 0.11 ^c^	1.88 ± 0.03 ^c^	4.87 ± 0.26	0.02
Atropine + Sample 2	2.60 ± 0.41 ^b^	2.07 ± 0.05	5.00 ± 0.15	0.01
Atropine + Sample 1	2.88 ± 0.03 ^b^	1.87 ± 0.05	4.90 ± 0.06	0.05
Verapamil	1.11 ± 0.04 ^a^	0.75 ± 0.02 ^a^	5.09 ± 0.06	0.02
Verapamil + Sample 2	2.37 ± 0.10 ^b^	2.00 ± 0.11	4.98 ± 0.12	0.04
Verapamil + Sample 1	2.90 ± 0.08 ^b^	1.94 ± 0.06	4.92 ± 0.21	0.03
Sample 2 + Verapamil	0.23 ± 0.03 ^d^	0.14 ± 0.02 ^d^	5.05 ± 0.04	0.03
Sample 1 + Verapamil	0.56 ± 0.07 ^d^	0.34 ± 0.03 ^d^	4.88 ± 0.19	0.01

Values are mean ± SD; Statistical significance: ᵃ—compared to basal spontaneous contractile activity; ᵇ—compared to individual application of *C. vulgaris* sample 1 or 2; ᶜ—compared to atropine; ᵈ—compared to individual application of verapamil (*p* < 0.05).

## Data Availability

The original contributions presented in the study are included in the article/Supplementary Materials. Further inquiries can be directed to the corresponding authors.

## Supplementary Materials

The following supporting information can be downloaded at https://www.mdpi.com/article/10.3390/foods14193319/s1, Table S1: Content of amino acids and organic acids in *C. vulgaris* samples; Table S2: Content of fatty acids, triterpenoids, and sterols in the *C. vulgaris* samples; Table S3: Carbohydrates identified in *C. vulgaris* samples.

## Abbreviations

The following abbreviations are used in this manuscript:

mTOR	Mammalian target of rapamycin
SM	Smooth muscle
NO	Nitric oxide
nNOS	Enzyme neuronal nitric oxide synthase
MP	Myenteric plexus

## References

[1] Wang C.A., Onyeaka H., Miri T., Soltani F. (2024). *Chlorella vulgaris* as a Food Substitute: Applications and Benefits in the Food Industry. J. Food Sci..

[2] Guil-Guerrero J.L., Prates J.A.M. (2025). Microalgae Bioactives for Functional Food Innovation and Health Promotion. Foods.

[3] Fekete G., Klátyik S., Sebők A., Dálnoki A.B., Takács A., Gulyás M., Czinkota I., Székács A., Gyuricza C., Aleksza L. (2024). Optimization of a *Chlorella vulgaris*-Based Carbon Sequestration Technique Using an Alkaline Medium of Wood Biomass Ash Extract. Water.

[4] Feng Y., Ge J., Show P.L., Song C., Wu L., Ma Z., Gao G. (2025). Using High CO_2_ Concentrations to Culture Microalgae for Lipid and Fatty Acid Production: Synthesis Based on a Meta-Analysis. Aquaculture.

[5] Mendes A.R., Spínola M.P., Lordelo M., Prates J.A.M. (2024). Advances in Bioprocess Engineering for Optimising *Chlorella vulgaris* Fermentation: Biotechnological Innovations and Applications. Foods.

[6] Canelli G., Tarnutzer C., Carpine R., Neutsch L., Bolten C.J., Dionisi F., Mathys A. (2020). Biochemical and Nutritional Evaluation of Chlorella and Auxenochlorella Biomasses Relevant for Food Application. Front. Nutr..

[7] Zheng X., Chen L., Yin L., Rao H., Zheng H., Xun C., Hao J. (2024). Application and Prospect of Microbial Food Chlorella. Heliyon.

[8] Mendes A.R., Spínola M.P., Lordelo M., Prates J.A.M. (2024). Chemical Compounds, Bioactivities, and Applications of *Chlorella vulgaris* in Food, Feed and Medicine. Appl. Sci..

[9] Abdel-Moatamed B.R., El-Fakhrany A.M.A., Elneairy N.A.A., Shaban M.M., Roby M.H.H. (2024). The Impact of *Chlorella vulgaris* Fortification on the Nutritional Composition and Quality Characteristics of Beef Burgers. Foods.

[10] Mtaki K., Kyewalyanga M.S., Mtolera M.S.P. (2020). Assessment of Antioxidant Contents and Free Radical-Scavenging Capacity of *Chlorella vulgaris* Cultivated in Low Cost Media. Appl. Sci..

[11] Ferdous U.T., Nurdin A., Ismail S., Yusof Z.N.B. (2023). Evaluation of the Antioxidant and Cytotoxic Activities of Crude Extracts from Marine *Chlorella* sp. *Biocatal*. Agric. Biotechnol..

[12] Tarsitano M., Ming C.L.C., Bennar L., Mahmodi H., Wyllie K., Idais D., Al Shamery W., Paolino D., Cox T.R., Kabakova I. (2025). Chlorella-Enriched Hydrogels Protect against Myocardial Damage and Reactive Oxygen Species Production in an In Vitro Ischemia/Reperfusion Model Using Cardiac Spheroids. Biofabrication.

[13] Elbarbary N.B., Abd El-Wahab R.A., Gad W.M., Zayed S., Basiony S., Darwish W.S., Abdallah M.S. (2025). Anti-Inflammatory, Antioxidant, and Immunomodulatory Effect of *Chlorella vulgaris* in Chicken Experimentally Infected with *Eimeria tenella* and *Clostridium perfringens*. Egypt. J. Vet. Sci..

[14] Capek P., Matulová M., Šutovská M., Barboríková J., Molitorisová M., Kazimierová I. (2020). *Chlorella vulgaris* α-L-Arabino-α-L-Rhamno-α,β-D-Galactan Structure and Mechanisms of Its Anti-Inflammatory and Anti-Remodelling Effects. Int. J. Biol. Macromol..

[15] Reis B., Ramos-Pinto L., Cunha S.A., Pintado M., da Silva J.L., Dias J., Conceição L., Matos E., Costas B. (2022). *Chlorella vulgaris* Extracts as Modulators of the Health Status and the Inflammatory Response of Gilthead Seabream Juveniles (*Sparus aurata*). Mar. Drugs.

[16] Samantaray M., Sahoo S., Sahoo D.P., Sethi G., Singh S., Lee H.-K., Pradhan B., Shin D. (2025). Computational Identification of Dual COX-1 and NIK Inhibitors from Marine Microalga *Chlorella vulgaris*. J. Genet. Eng. Biotechnol..

[17] Jo S.W., Velankanni P., Cam N.D.T., Nguyen C.H.B., Jeon J.Y., Kim B.R., Lee D.H., Lee C.G., Park J.S. (2025). Chemical Profiling and Immune-Stimulating Activity of Solvent Fractions Derived from Dietary Chlorella. J. Microbiol. Biotechnol..

[18] Jeong K.-M., Choi Y.-J., Jeong H.-J. (2025). Immunomodulatory and Energy-Enhancing Effects of Modified SOUL-Tang. CellMed.

[19] Ewert A.M., McMenamin A., Adjaye D., Rainey V., Ricigliano V. (2025). Microalgae Functional Feed Additives Strengthen Immunity and Increase Longevity in Honey Bees. J. Invertebr. Pathol..

[20] Ferdous U.T., Khan S.A., Shakoor A., Uddin S., Nazarudin M.F., Zia A.W. (2025). *Chlorella* spp. as an Emerging Source for Anticancer Remedy and Nutraceuticals: An Advance Study. Food Rev. Int..

[21] Almutairi L.A., Abdelghaffar E.G., Hafney H.A., Ebaid H.M., Alkhodair S.A., Shaalan A.A.M., El-Hak H.N.G. (2025). Protective Impacts of *Chlorella vulgaris* on Cisplatin-Induced Toxicity in Liver, Kidney, and Spleen of Rats: Role of Oxidative Stress, Inflammation, and Nrf2 Modulation. Life.

[22] Mustafa G., Islam M., Ahmad I., Shakir H.A., Khan M., Irfan M. (2025). Harnessing the Anticancer Potential of Algae: A Comprehensive Review. ChemBioEng Rev..

[23] Abo-Shady A.M., Gheda S.F., Ismail G.A., Cotas J., Pereira L., Abdel-Karim O.H. (2023). Antioxidant and Antidiabetic Activity of Algae. Life.

[24] Barghchi H., Dehnavi Z., Nattagh-Eshtivani E., Alwaily E.R., Almulla A.F., Kareem A.K., Barati M., Ranjbar G., Mohammadzadeh A., Rahimi P. (2023). The Effects of *Chlorella vulgaris* on Cardiovascular Risk Factors: A Comprehensive Review on Putative Molecular Mechanisms. Biomed. Pharmacother..

[25] Kaeoboon S., Songserm R., Suksungworn R., Duangsrisai S., Sanevas N. (2025). In Vitro Antioxidant Potential, Antidiabetic Activities, and GC-MS Analysis of Lipid Extracts of Chlorella Microalgae. BioTech.

[26] Dimopoulou M., Kolonas A., Stagos D., Gortzi O. (2025). A Review of the Sustainability, Chemical Composition, Bioactive Compounds, Antioxidant and Antidiabetic Activity, Neuroprotective Properties, and Health Benefits of Microalgae. Biomass.

[27] Shah Z., Iqbal A., Badshah S.L., Rauf K., Nabi G., Khan H., Ullah A., Ali S., Bibi S., Haider S. (2025). Macroalgae Polysaccharides Enhance Brain Health by Mitigating Scopolamine-Induced Oxidative Stress and Inflammation via Nrf2/TLR4/NF-κB Pathways. J. Neuroimmune Pharmacol..

[28] Gochhi M., Dash P., Kar B., Pradhan D., Halder J., Das C., Rai V.K., Rout S.K., Ghosh G., Rath G. (2025). Anti-Hypertensive Function of Plant-Derived Bioactive Peptides: A Review. Curr. Pharm. Des..

[29] Velankanni P., Go S.H., Jin J.B., Park J.S., Park S., Lee S.B., Kwon H.K., Pan C.H., Cha K.H., Lee C.G. (2023). *Chlorella vulgaris* Modulates Gut Microbiota and Induces Regulatory T Cells to Alleviate Colitis in Mice. Nutrients.

[30] Bañares C., Paterson S., Gómez-Garre D., Ortega-Hernández A., Sánchez-González S., Cueva C., de la Fuente M.Á., Hernández-Ledesma B., Gómez-Cortés P. (2025). Modulation of Gut Microbiota and Short-Chain Fatty Acid Production by Simulated Gastrointestinal Digests from Microalga *Chlorella vulgaris*. Int. J. Mol. Sci..

[31] Brizzi A., Rispoli R.M., Autore G., Marzocco S. (2025). Anti-Inflammatory Effects of Algae-Derived Biomolecules in Gut Health: A Review. Int. J. Mol. Sci..

[32] Nishimoto Y., Nomaguchi T., Mori Y., Ito M., Nakamura Y., Fujishima M., Murakami S., Yamada T., Fukuda S. (2021). The Nutritional Efficacy of Chlorella Supplementation Depends on the Individual Gut Environment: A Randomised Control Study. Front. Nutr..

[33] Feng S., Hernández-Olivas E., Sahin A.W., Giblin L., Brodkorb A. (2025). Semi-Dynamic In Vitro Digestion of Honey *Chlorella vulgaris* Reveals Biochemical and Structural Insights during Gastrointestinal Transit. Food Res. Int..

[34] Zholos A.V., Melnyk M.I., Dryn D.O. (2024). Molecular Mechanisms of Cholinergic Neurotransmission in Visceral Smooth Muscles with a Focus on Receptor-Operated TRPC4 Channel and Impairment of Gastrointestinal Motility by General Anaesthetics and Anxiolytics. Neuropharmacology.

[35] Sampaio Moura N., Schledwitz A., Alizadeh M., Kodan A., Njei L.-P., Raufman J.-P. (2024). Cholinergic Mechanisms in Gastrointestinal Neoplasia. Int. J. Mol. Sci..

[36] Martínez-Pérez E.F., Juárez Z.N., Hernández L.R., Bach H. (2018). Natural Antispasmodics: Source, Stereochemical Configuration, and Biological Activity. Biomed Res. Int..

[37] Aguirre-Crespo F.J., Aragón-Gastélum J.L., Gutiérrez-Alcántara E.J., Zamora-Crescencio P., Gómez-Galicia D.L., Alatriste-Kurzel D.R., Alvarez G., Hernández-Núñez E. (2024). β-Sitosterol Mediates Gastrointestinal Smooth Muscle Relaxation Induced by *Coccoloba uvifera* via Muscarinic Acetylcholine Receptor Subtype 3. Sci. Pharm..

[38] Xu Y., Zhu R., Chen T., Guo Z., Peng T., Zhao L. (2025). Antispasmodic Effects and Potential Mechanism of the Traditional Chinese Medicine Shaoyao–Gancao Decoction on Intestinal Spasm in Rats. Pharmacol. Res. Mod. Chin. Med..

[39] (2009). Particle Size Analysis—Laser Diffraction Methods.

[40] Dincheva I., Badjakov I., Georgiev V., Semerdjieva I., Vrancheva R., Ivanov I., Pavlov A. (2025). Comprehensive GC-MS Characterization and Histochemical Assessment of Various Parts of Three *Colchicum* Species from Bulgarian Flora. Plants.

[41] (2008). NIST Standard Reference Database 1A: NIST/EPA/NIH Mass Spectral Library (NIST 08) and NIST Mass Spectral Search Program (Version 20f) Manual.

[42] Hummel J., Strehmel N., Selbig J., Walther D., Kopka J. (2010). Decision Tree-Supported Substructure Prediction of Metabolites from GC-MS Profiles. Metabolomics.

[43] Milusheva M., Todorova M., Gledacheva V., Stefanova I., Feizi-Dehnayebi M., Pencheva M., Nedialkov P., Tumbarski Y., Yanakieva V., Tsoneva S. (2023). Novel Anthranilic Acid Hybrids—An Alternative Weapon against Inflammatory Diseases. Pharmaceuticals.

[44] Milusheva M., Gledacheva V., Stefanova I., Feizi-Dehnayebi M., Mihaylova R., Nedialkov P., Cherneva E., Tumbarski Y., Tsoneva S., Todorova M. (2023). Synthesis, Molecular Docking, and Biological Evaluation of a Novel Anthranilic Acid Hybrid and Its Diamides as Antispasmodics. Int. J. Mol. Sci..

[45] Shu Y., Li J., Yang X., Dong X., Wang X. (2019). Effect of Particle Size on the Bioaccessibility of Polyphenols and Polysaccharides in Green Tea Powder and Its Antioxidant Activity after Simulated Human Digestion. J. Food Sci. Technol..

[46] Wang E., Yin Z., Zeng M. (2022). Microalgae as a Promising Structure Ingredient in Food: Obtained by Simple Thermal and High-Speed Shearing Homogenization. Food Hydrocoll..

[47] Pantami H.A., Ahamad Bustamam M.S., Lee S.Y., Ismail I.S., Mohd Faudzi S.M., Nakakuni M., Shaari K. (2020). Comprehensive GC-MS and LC-MS/MS Metabolite Profiling of *Chlorella vulgaris*. Mar. Drugs.

[48] Krivina E., Degtyaryov E., Tebina E., Temraleeva A., Savchenko T. (2024). Comparative Analysis of the Fatty Acid Profiles of Selected Representatives of Chlorella-Clade to Evaluate Their Biotechnological Potential. Int. J. Plant Biol..

[49] Kotlyarov S., Kotlyarova A. (2022). Involvement of Fatty Acids and Their Metabolites in the Development of Inflammation in Atherosclerosis. Int. J. Mol. Sci..

[50] Zahid S., Malik A., Waqar S., Zahid F., Tariq N., Khawaja A.I., Safir W., Gulzar F., Iqbal J., Ali Q. (2023). Countenance and Implication of β-Sitosterol, β-Amyrin and Epiafzelechin in Nickel-Exposed Rats: In-Silico and In-Vivo Approach. Sci. Rep..

[51] Li J., Chen J., An L., Yuan X., Yao L. (2020). Polyol and Sugar Osmolytes Can Shorten Protein Hydrogen Bonds to Modulate Function. Commun. Biol..

[52] Santa-María C., López-Enríquez S., Montserrat-de la Paz S., Geniz I., Reyes-Quiroz M.E., Moreno M., Palomares F., Sobrino F., Alba G. (2023). Update on Anti-Inflammatory Molecular Mechanisms Induced by Oleic Acid. Nutrients.

[53] Vieira A.P., Lelis C.A., Ochioni A.C., Rosário D.K.A., Rosario I.L.S., Vieira I.R.S., Carvalho A.P.A., Janeiro J.M., da Costa M.P., Lima F.R.S. (2024). Estimating the Therapeutic Potential of NSAIDs and Linoleic Acid-Isomers Supplementation against Neuroinflammation. Biomed. Pharmacother..

[54] Seufert A.L., Napier B.A. (2023). A New Frontier for Fat: Dietary Palmitic Acid Induces Innate Immune Memory. Immunometabolism.

[55] Chaudhari S.P., Baviskar D.T. (2021). Anti-Inflammatory Activity of *Chlorella vulgaris* in Experimental Models of Rats. Int. J. Pharm. Investig..

[56] Savvidou M.G., Georgiopoulou I., Antoniou N., Tzima S., Kontou M., Louli V., Fatouros C., Magoulas K., Kolisis F.N. (2023). Extracts from *Chlorella vulgaris* Protect Mesenchymal Stromal Cells from Oxidative Stress Induced by Hydrogen Peroxide. Plants.

[57] Nunes C.R., Barreto Arantes M., Menezes de Faria Pereira S., Leandro da Cruz L., de Souza Passos M., Pereira de Moraes L., Vieira I.J.C., Barros de Oliveira D. (2020). Plants as Sources of Anti-Inflammatory Agents. Molecules.

[58] Yan D., Ye S., He Y., Wang S., Xiao Y., Xiang X., Deng M., Luo W., Chen X., Wang X. (2023). Fatty Acids and Lipid Mediators in Inflammatory Bowel Disease: From Mechanism to Treatment. Front. Immunol..

[59] Hossain R., Kim K.I., Jin F., Lee H.J., Lee C.J. (2022). Betulin, an Anti-Inflammatory Triterpenoid Compound, Regulates MUC5AC Mucin Gene Expression through NF-κB Signaling in Human Airway Epithelial Cells. Biomol. Ther..

[60] Park J.H., Kwon J.G., Kim S.J., Song D.K., Lee S.G., Kim E.S., Cho K.B., Jang B.I., Kim D.H., Sin J.-I. (2014). Alterations of Colonic Contractility in an Interleukin-10 Knockout Mouse Model of Inflammatory Bowel Disease. J. Neurogastroenterol. Motil..

[61] Ohama T., Hori M., Ozaki H. (2007). Mechanism of Abnormal Intestinal Motility in Inflammatory Bowel Disease: How Smooth Muscle Contraction Is Reduced?. J. Smooth Muscle Res..

[62] Malykhina A.P., Akbarali H.I. (2004). Inflammation-Induced “Channelopathies” in the Gastrointestinal Smooth Muscle. Cell Biochem. Biophys..

[63] Gougeon P.-Y., Lourenssen S., Han T.Y., Nair D.G., Ropeleski M.J., Blennerhassett M.G. (2013). The Pro-Inflammatory Cytokines IL-1β and TNFα Are Neurotrophic for Enteric Neurons. J. Neurosci..

[64] Hąc-Wydro K., Wydro P. (2007). The Influence of Fatty Acids on Model Cholesterol/Phospholipid Membranes. Chem. Phys. Lipids.

[65] Ruiz M., Palmgren H., Henricsson M., Devkota R., Jaiswal H., Maresca M., Bohlooly Y.M., Peng X.-R., Borén J., Pilon M. (2021). Extensive Transcription Mis-Regulation and Membrane Defects in AdipoR2-Deficient Cells Challenged with Saturated Fatty Acids. Biochim. Biophys. Acta-Mol. Cell Biol. Lipids.

[66] Harayama T., Antonny B. (2023). Beyond Fluidity: The Role of Lipid Unsaturation in Membrane Function. Cold Spring Harb. Perspect. Biol..

[67] Mondal D., Dutta R., Banerjee P., Mukherjee D., Maiti T.K., Sarkar N. (2019). Modulation of Membrane Fluidity Performed on Model Phospholipid Membrane and Live Cell Membrane: Revealing through Spatiotemporal Approaches of FLIM, FAIM, and TRFS. Anal. Chem..

[68] Tanahashi Y., Komori S., Matsuyama H., Kitazawa T., Unno T. (2021). Functions of Muscarinic Receptor Subtypes in Gastrointestinal Smooth Muscle: A Review of Studies with Receptor-Knockout Mice. Int. J. Mol. Sci..

[69] Saponaro A., Lolicato M. (2022). Editorial: The Key Role of Lipids in the Regulation of Ion Channels. Front. Physiol..

[70] Ali O., Szabó A. (2023). Review of Eukaryote Cellular Membrane Lipid Composition, with Special Attention to the Fatty Acids. Int. J. Mol. Sci..

[71] Tao Y., Wu Y., Jiang C., Wang Q., Geng X., Chen L., Zhou S., Wang X., Han M., Du D. (2023). Saturated Fatty Acid Promotes Calcification via Suppressing SIRT6 Expression in Vascular Smooth Muscle Cells. J. Hypertens..

[72] Santos F.A., Carvalho K.M.M.B., Batista-Lima F.J., Nunes P.I.G., Viana A.F.S.C., de Carvalho Almeida da Silva A.A., da Cruz Fonseca S.G., Chaves M.H., Rao V.S., Magalhães P.J.C. (2017). The Triterpenoid α,β-Amyrin Prevents the Impaired Aortic Vascular Reactivity in High-Fat Diet-Induced Obese Mice. Naunyn Schmiedebergs Arch. Pharmacol..

[73] Hernández E.A.G., Galindo G.C., Chávez R.S.M., Moreno P.C., Barajas M.I., Duque T.E.V., Antúnez A.P.H., Mondragón L.D.V., Guerrero G.A.M., Cobos D.S. (2025). Antioxidant, Antidiabetic, and Vasorelaxant Effects of Ethanolic Extract from the Seeds of *Swietenia humilis*. Int. J. Mol. Sci..

[74] Aida K., Mita M., Ishii-Nozawa R. (2024). Difference in Contractile Mechanisms between the Early and Sustained Components of Ionomycin-Induced Contraction in Rat Caudal Arterial Smooth Muscle. Biol. Pharm. Bull..

[75] Ji R.R., Nackley A., Huh Y., Terrando N., Maixner W. (2018). Neuroinflammation and Central Sensitization in Chronic and Widespread Pain. Anesthesiology.

[76] Mailhot B., Christin M., Tessandier N., Sotoudeh C., Bretheau F., Turmel R., Pellerin È., Wang F., Bories C., Joly-Beauparlant C. (2020). Neuronal Interleukin-1 Receptors Mediate Pain in Chronic Inflammatory Diseases. J. Exp. Med..

[77] Dinarello C.A. (2011). A Clinical Perspective of IL-1β as the Gatekeeper of Inflammation. Eur. J. Immunol..

[78] Snodgrass R.G., Huang S., Choi I.W., Rutledge J.C., Hwang D.H. (2013). Inflammasome-Mediated Secretion of IL-1β in Human Monocytes through TLR2 Activation; Modulation by Dietary Fatty Acids. J. Immunol..

[79] Korbecki J., Bajdak-Rusinek K. (2019). The Effect of Palmitic Acid on Inflammatory Response in Macrophages: An Overview of Molecular Mechanisms. Inflamm. Res..

[80] Triantafilou M., Miyake K., Golenbock D.T., Triantafilou K. (2002). Mediators of Innate Immune Recognition of Bacteria Concentrate in Lipid Rafts and Facilitate Lipopolysaccharide-Induced Cell Activation. J. Cell Sci..

[81] Bodur M., Yilmaz B., Ağagündüz D., Ozogul Y. (2025). Immunomodulatory Effects of Omega-3 Fatty Acids: Mechanistic Insights and Health Implications. Mol. Nutr. Food Res..

[82] Ren C., Jin J., Hu W., Chen Q., Yang J., Wu Y., Zhou Y., Sun L., Gao W., Zhang X. (2021). Betulin Alleviates the Inflammatory Response in Mouse Chondrocytes and Ameliorates Osteoarthritis via AKT/Nrf2/HO-1/NF-κB Axis. Front. Pharmacol..

[83] Su C.-H., Lin C.-Y., Tsai C.-H., Lee H.-P., Lo L.-C., Huang W.-C., Wu Y.-C., Hsieh C.-L., Tang C.-H. (2021). Betulin Suppresses TNF-α and IL-1β Production in Osteoarthritis Synovial Fibroblasts by Inhibiting the MEK/ERK/NF-κB Pathway. J. Funct. Foods.

[84] Sharkey K.A., Mawe G.M. (2023). The Enteric Nervous System. Physiol. Rev..

[85] Kim K., Lee I., Gu W., Hyam S.R., Kim D. (2014). β-Sitosterol Attenuates High-Fat Diet-Induced Intestinal Inflammation in Mice by Inhibiting the Binding of Lipopolysaccharide to Toll-like Receptor 4 in the NF-ΚB Pathway. Mol. Nutr. Food Res..

[86] Reichardt F., Chassaing B., Nezami B.G., Li G., Tabatabavakili S., Mwangi S., Uppal K., Liang B., Vijay-Kumar M., Jones D. (2017). Western Diet Induces Colonic Nitrergic Myenteric Neuropathy and Dysmotility in Mice via Saturated Fatty Acid- and Lipopolysaccharide-Induced TLR4 Signalling. J. Physiol..

[87] Rocha D.M., Caldas A.P., Oliveira L.L., Bressan J., Hermsdorff H.H. (2016). Saturated Fatty Acids Trigger TLR4-Mediated Inflammatory Response. Atherosclerosis.

[88] Coniglio S., Shumskaya M., Vassiliou E. (2023). Unsaturated Fatty Acids and Their Immunomodulatory Properties. Biology.

