# Supplementary material for: Metabolite Signatures and Particle Size as Determinants of Anti-Inflammatory and Gastrointestinal Smooth Muscle Modulation by Chlorella vulgaris
Source: Foods. 2025 Sep 25;14(19):3319. doi: 10.3390/foods14193319 (PMC12524018; doi:10.3390/foods14193319)
Supplement: Supplementary file 1 [file foods-14-03319-s001.zip › foods-3867034-supplementary.pdf]

Table S1. Content of amino acids and organic acids in *C. vulgaris* samples.

**Compound**	**RT**	**RI**	**Sample 1 (India)**	**Sample 2 (UK)**
			**Content ± SD, mg/g extract**	
Essential amino acids				
L-Valine	4.62	1208	0.87 ± 0.09^{a}	0.23 ± 0.02^{b}
L-Leucine	6.07	1274	1.28 ± 0.13^{b}	2.46 ± 0.26^{a}
L-Isoleucine	6.20	1286	0.68 ± 0.07^{b}	1.23 ± 0.13^{a}
L-Threonine	7.84	1376	1.07 ± 0.11^{a}	0.73 ± 0.08^{b}
Non-essential amino acids				
L-Glutamic acid	10.43	1609	4.74 ± 0.50^{a}	1.97 ± 0.21^{b}
Alanine	4.50	1097	1.87 ± 0.20^{a}	0.63 ± 0.07^{b}
Conditional non-essential amino acids				
L-Aspartic acid	8.78	1502	11.99 ± 1.26^{a}	0.43 ± 0.05^{b}
Proline	6.28	1292	3.81 ± 0.40^{a}	0.39 ± 0.04^{b}
Pyroglutamic acid	9.35	1517	9.53 ± 1.00^{a}	3.22 ± 0.34^{b}
L-Serine	7.12	1351	0.74 ± 0.08^{a}	0.30 ± 0.03^{b}
Organic acids				
Malic acid	8.47	1475	7.08 ± 0.75^{a}	1.44 ± 0.15^{b}
Succinic acid	6.35	1307	1.74 ± 0.18^{a}	0.57 ± 0.06^{b}
Fumaric acid	6.51	1345	1.55 ± 0.16^{a}	0.55 ± 0.06^{b}
Salicylic acid	8.91	1505	nd	2.65 ± 0.28^{a}

* Compounds were identified by comparison with authentic standards (when available) or by NIST/Wiley MS library matching (≥90% similarity) combined with retention index (RI) agreement within ±2% of literature/standards. Content values are expressed as mean ± SD (n = 3). Means in a row with different superscript letters (a–b) differ significantly ($p < 0.05$; Duncan's test); nd – not detected.

Table S2. Content of fatty acids, triterpenoids, and sterols in the *C. vulgaris* samples.

Compound	RT	RI	Sample 1 (India)	Sample 2 (UK)
			Content ± SD, mg/g extract	
Fatty acids				
Lauric acid (C12:0)	19.05	1524	6.97 ± 0.86^{a}	3.27 ± 0.40^{b}
Myristic acid (C14:0)	22.67	1728	9.04 ± 1.12^{a}	4.59 ± 0.57^{b}
Palmitic acid (C16:0)	29.80	1940	8.99 ± 1.11^{b}	19.13 ± 2.36^{a}
Margaric acid (C17:0)	27.62	1826	16.88 ± 2.08^{a}	8.34 ± 1.03^{b}
Stearic acid (C18:0)	7.84	1376	1.92 ± 0.24^{a}	2.28 ± 0.28^{a}
Arachidic acid (C20:0)	35.01	2341	3.86 ± 0.48^{a}	2.63 ± 0.33^{b}
Behenic acid (C22:0)	38.84	2542	10.46 ± 1.29^{a}	8.04 ± 0.99^{b}
Lignoceric acid (C24:0)	42.21	2739	1.88 ± 0.23^{a}	1.75 ± 0.22^{a}
Cerotic acid (C26:0)	46.33	2938	20.82 ± 2.57^{a}	3.37 ± 0.42^{b}
Oleic acid (C18:1)	32.86	2102	2.18 ± 0.27^{b}	18.74 ± 2.31^{a}
Linoleic acid (C18:2)	32.75	2096	10.23 ± 1.26^{b}	29.45 ± 3.64^{a}
Triterpenoid Alcohols and Phytosterols				
Betulin	6.35	1307	nd	2.25 ± 0.28^{a}
β-Sitosterol	6.51	1345	4.47 ± 0.55^{a}	2.63 ± 0.32^{b}
β-Amyrin	49.41	3334	4.07 ± 0.50^{a}	2.04 ± 0.25^{b}

* Compounds were identified by comparison with authentic standards (when available) or by NIST/Wiley MS library matching (≥90% similarity) combined with retention index (RI) agreement within ±2% of literature/standards. Content values are expressed as mean ± SD (n = 3). Means in a row with different superscript letters (a–b) differ significantly ($p < 0.05$; Duncan's test); nd – not detected.

Table S3. Carbohydrates identified in *C. vulgaris* samples.

Compound	RT	RI	Sample 1 (India)	Sample 2 (UK)
			Content ± SD, mg/g extract	
Mono- and disaccharides				
Fructose	12.48	1855	0.95 ± 0.10^{a}	0.74 ± 0.08^{a}
Glucose	13.22	1901	1.51 ± 0.16^{b}	4.02 ± 0.42^{a}
Sucrose	24.3	2655	3.81 ± 0.40^{b}	8.04 ± 0.85^{a}
Mannose-6-phosphate methoxyamine	19.98	2296	3.27 ± 0.34^{b}	22.79 ± 2.40^{a}
Sugar alcohols				
Glucitol	13.81	1933	20.92 ± 2.20^{a}	0.96 ± 0.10^{b}

* Compounds were identified by comparison with authentic standards (when available) or by NIST/Wiley MS library matching (⩾90% similarity) combined with retention index (RI) agreement within ±2% of literature/standards. Content values are expressed as mean ± SD (n = 3). Means in a row with different superscript letters (a–b) differ significantly ($p < 0.05$; Duncan's test).
